# Proximity-Based Labeling Identifies MHC Class II and CD37 as B Cell Receptor–Proximal Proteins with Immunological Functions

**DOI:** 10.4049/immunohorizons.2400014

**Published:** 2024-04-16

**Authors:** Sean Hoeger, Lisa A. Drake, James R. Drake

**Affiliations:** Department of Immunology and Microbial Disease, Albany Medical College, Albany, NY

## Abstract

The BCR allows for Ag-driven B cell activation and subsequent Ag endocytosis, processing, and presentation to recruit T cell help. Core drivers of BCR signaling and endocytosis are motifs within the receptor’s cytoplasmic tail (primarily CD79). However, BCR function can be tuned by other proximal cellular elements, such as CD20 and membrane lipid microdomains. To identify additional proteins that could modulate BCR function, we used a proximity-based biotinylation technique paired with mass spectrometry to identify molecular neighbors of the murine IgM BCR. Those neighbors include MHC class II molecules, integrins, various transporters, and membrane microdomain proteins. Class II molecules, some of which are invariant chain–associated nascent class II, are a readily detected BCR neighbor. This finding is consistent with reports of BCR–class II association within intracellular compartments. The BCR is also in close proximity to multiple proteins involved in the formation of membrane microdomains, including CD37, raftlin, and Ig superfamily member 8. Known defects in T cell–dependent humoral immunity in CD37 knockout mice suggest a role for CD37 in BCR function. In line with this notion, CRISPR-based knockout of CD37 expression in a B cell line heightens BCR signaling, slows BCR endocytosis, and tempers formation of peptide–class II complexes. These results indicate that BCR molecular neighbors can impact membrane-mediated BCR functions. Overall, a proximity-based labeling technique allowed for identification of multiple previously unknown BCR molecular neighbors, including the tetraspanin protein CD37, which can modulate BCR function.

## Introduction

Binding of Ag to the BCR expressed on the surface of B lymphocytes is the molecular event underlying clonal selection, which drives initiation of an Ag-specific humoral immune response. Ag binding to the BCR results in both signaling events that trigger B cell activation and BCR-mediated Ag endocytosis and processing, resulting in formation of complexes of Ag-derived peptide and MHC class II (MHCII) molecules. These peptide–MHCII complexes are then expressed on the surface of the B cell to recruit T cell help, resulting in generation of a robust humoral immune response.

The BCR is composed of two subunits: an Ag-binding subunit composed of IgH/L chains, which is paired with a signaling subunit composed of CD79A and CD79B (CD79). The CD79 cytoplasmic tails contain both ITAMs to drive BCR signaling (1) and tyrosine-based endocytosis motifs to drive internalization of Ag–BCR complexes (2, 3). Although the BCR contains all of the elements necessary for BCR signaling and endocytosis, these BCR activities can be significantly impacted by neighboring cellular components (CCs). For example, membrane microdomains known as lipid rafts, as well as membrane proteins such as CD20 and FcγRII, can profoundly impact BCR signaling (4–6). Thus, a complete understanding of BCR function requires an understanding of the full spectrum of BCR molecular neighbors that could impact BCR behavior.

Some techniques that allow for the analysis of molecular proximity in intact cells include Förster’s resonance energy transfer (FRET), bioluminescence resonance energy transfer, and PCR-based proximity ligation assay. However, in each of these cases, the identity of the two target proteins must be known so appropriate probes may be developed. More recently, discovery style approaches such as selective proteomic proximity labeling using tyramide (SPPLAT) and APEX2 (discussed later) have been used to search for previously unknown molecular neighbors.

SPPLAT is an approach where HRP is targeted to a protein of interest and then used to catalyze formation of a short-lived reactive form of biotin to tag neighboring molecules. Biotin-tagged molecules are then isolated with streptavidin (SA)-beads and identified by mass spectrometry (MS). The use of this technique in studying the BCR was pioneered by the Jackson laboratory (7). They used anti–IgM-HRP to target HRP to cell-surface BCR molecules of chicken DT-40 B cells and SPPLAT to label the receptor’s molecular neighbors. Using this approach, they demonstrate that integrins and the chicken B lymphocyte allotypic marker chB6 are major BCR molecular neighbors. However, chicken DT-40 B cells do not express MHCII molecules, limiting the ability of the authors to probe BCR proximity to molecules involved in Ag processing/presentation.

APEX2 involves the use of engineered ascorbate peroxidase linked to a protein of interest to biotin-tag neighboring molecules. Biotin-tagged molecules are again harvested with SA-beads and identified by MS. The use of APEX2 in studying the BCR has been pioneered by the Mattila laboratory (8). They engineered a form of APEX2 targeted to the inner face of lipid rafts via an lck N-terminal peptide, and used it to study lipid raft–mediated BCR signaling in the murine A20 B cell line. These studies suggest a previously unappreciated role for SUMOylation in BCR signaling. They also reveal a low/variable level of MHCII raft association, as well as BCR-triggered raft association of the Vti1b SNARE protein that has been linked to the trafficking of the MHCII chaperone invariant chain (Ii) (9). The Vti1b results were further investigated, but the authors could not identify a role for this SNARE in BCR function (10).

The goal of this study was to use BCR-directed SPPLAT labeling (BCR-SPPLAT) with a B cell that has an intact MHCII Ag processing/presentation pathway to identify and investigate BCR molecular neighbors that may impact BCR-mediated Ag processing and presentation. This approach led to the identification of ∼100 BCR molecular neighbors, including MHCII molecules and the tetraspanin CD37. Ablation of CD37 expression results in changes in BCR signaling, endocytosis, and formation of peptide–class II complexes. Future analysis of other identified BCR molecular neighbors will likely reveal additional proteins that can regulate BCR function.

## Materials and Methods

### Cells

The K46µ, 1D6 A10, and A20µWT B cell lines were cultured as previously reported (11). Splenocytes were isolated from C57BL//6 mice (purchased from Jackson Laboratories) by Ficoll-Paque as previously reported (12). The animal protocol (euthanasia and subsequent harvesting of the spleen) was reviewed and approved by the Albany Medical College Institutional Animal Care and Use Committee.

### Reagents

The reagents used in the this study were sulfo-NHS-LC-biotin (21335; Thermo Scientific), anti–CD37-PE (146204; BioLegend), anti–I-A^d^-FITC (AMS-32.1-FITC, 553547; BD Biosciences), anti–I-A^k^-FITC (10-3.6-FITC, 553547; BD Biosciences), anti–IgM^a^-PE (553517; BD Biosciences), 1630a rabbit anti–I-A β-chain cytoplasmic tail (13), and 34-5-3S (2025-4; Southern Biotech).

### BCR-SPPLAT labeling

Cells were BCR-SPPLAT labeled following the published protocol of Li et al. (7). In brief, anti–BCR-HRP (1:100 dilution of either rat anti-mouse IgM-HRP mAb [1140-05; Southern Biotech] or goat anti-human IgM-HRP [2020-05; Southern Biotech]) was bound to cells for 20 min on ice. Cells were washed, sometimes incubated at 37°C, then resuspended in 50 mM Tris (pH 7.5), 0.03% H_2_O_2_, and 80 µg/ml tyramide-biotin for 10 min at room temperature (SPPLAT labeling). H_2_O_2_ was then destroyed by treatment with 10 U/ml catalase for 10 min. Samples were then lysed at 10^7^ cells/ml in radioimmunoprecipitation assay (RIPA) buffer (50 mM Tris [pH 7.5], 150 mM NaCl, 5 mM EDTA, 0.5% [w/v] deoxycholate, 1% [v/v] NP-40 substitute [Igelap CA-630 (octylphenoxy)polyoxyethanol, CAS Registry Number 68987-90-6; Catalog No. 19628; USB Corporation]) with protease inhibitors.

### Pull-down conditions

Cleared whole-cell lysate (WCL) was immunoprecipitated with either anti–class II and protein A–agarose beads (22812; Thermo) or SA-agarose beads (20353; Thermo). Samples were boiled in 1× SDS-PAGE sample buffer and stored at −20°C until analysis.

### Tyramide-biotin synthesis

Tyramide-biotin was synthesized as previously reported (7). In brief, 3.73 mg of NHS-LC-biotin (21336; Thermo) in 50 µl of DMSO and 1.55 mg of tyramine hydrochloride (T2879, 10% molar excess; Sigma) in 50 µl of DMSO were combined, and then 2 ml of 50 mM borate (pH 8.8) was added. The sample was incubated overnight at room temperature in the dark. Aliquot samples were then stored at −20°C.

### Mass spectrometry

Samples of biotin-tagged proteins captured on SA-agarose beads (20353; Thermo) were boiled in 1× SDS-PAGE reducing sample buffer, frozen at −20°C, and then shipped to MS Bioworks (Ann Arbor, MI) on dry ice. The samples were subject to immunoprecipitation (IP) profiling analysis (MSB23). The results of three independent experiments were combined and analyzed in Scaffold Viewer Version 5. The results were also exported to Excel for additional analysis.

### CD37Δ

We purchased three CD37-targeting transEDIT CRISPR vectors from Transomics (TEVM-1263705, 11966563, 1129421), each of which encode a CD37-directed guide RNA, CRISPR, and puromycin resistance (the sequence of the target gene–specific guide RNAs is proprietary information of Transomics). Plasmids were introduced into 1D6 A10 cells by electroporation. Bulk cultures of transfected cells were grown in the presence of 10 µg/ml puromycin and cells from the surviving population cloned by limiting dilution. Clones were screened for CD37 expression by staining with anti-CD37 (and anti–I-A^d^ as a control) and CD37Δ clones selected for further analysis. The 2D10 and 3E3 clones arose from cells transfected with the TEVM-1129421 plasmid, and 1C2 and 2C11 clones from cells transfected with the TEVM-11966563 plasmid. CD37Δ clones were also screened by PCR using CD37 primers (forward 5′-GCCTGAGCTATGGACACCTG-3′ and reverse 5′-CGTCATGAAGCCGAGCTCAA-3′) and primers for the β-chain of I-A as a loading control (forward 5′-AGCTAAGCTTGTTATGGCTCTGCAGATCCCCAG-3′ and reverse 5′-AGCTTCTAGATCACTGCAGGAGCCCTGCTG-3′). RNA was converted to cDNA using random hexamers as primers and 30-cycle PCR of the cDNA run using the earlier primers and AccuPrime Taq DNA polymerase.

### Flow cytometry

Samples were analyzed on a BD FACSymphony flow cytometer controlled by FACSDiva software version 8.0. Resulting data were analyzed with FlowJo software v9.9.4. Samples were gated for single cells by forward scatter/side scatter (FCS/SSC) before analysis of fluorescent intensity.

### Calcium signaling

As previously reported (14), B cells were loaded with Fluo-3 and Fura Red in the presence of probenecid (to prevent dye efflux). After resting for 1–2 h at room temperature in the dark, the cells were stimulated, and the Fluo-3/Fura Red levels were monitored by flow cytometry. The ratio of Fluo-3 to Fura Red was used as a measure of relative intracellular calcium.

### Ag processing/presentation

B cells were cultured overnight at 5 × 10^5^ viable cells/ml in media containing the indicated level of hen egg lysozyme (HEL; L 6876; Sigma) or NP-HEL (sc-396474; Santa Cruz). Cells were then harvested, washed, and stained with C4H3 to detect HEL_46–61_–I-A^k^ peptide–class II complexes (12, 15), followed by secondary Ab (anti-rat IgG_2b_-FITC, 556999; BD Biosciences). Cells were then analyzed by flow cytometry. For each experiment, the level of C4H3 staining of cells pulsed with 50 µM HEL was set to a value of 1.00, and all other values were normalized to that level. Cells exposed to HEL for fluid-phase Ag processing were *not* stimulated with anti-BCR Ab because differences in BCR signaling between wild-type and CD37Δ cells (Fig. 6B) might differentially impact this reference pathway of Ag processing/presentation.

## Results

### Proximity-based biotinylation identifies a set of BCR molecular neighbors

**FIGURE 1.**
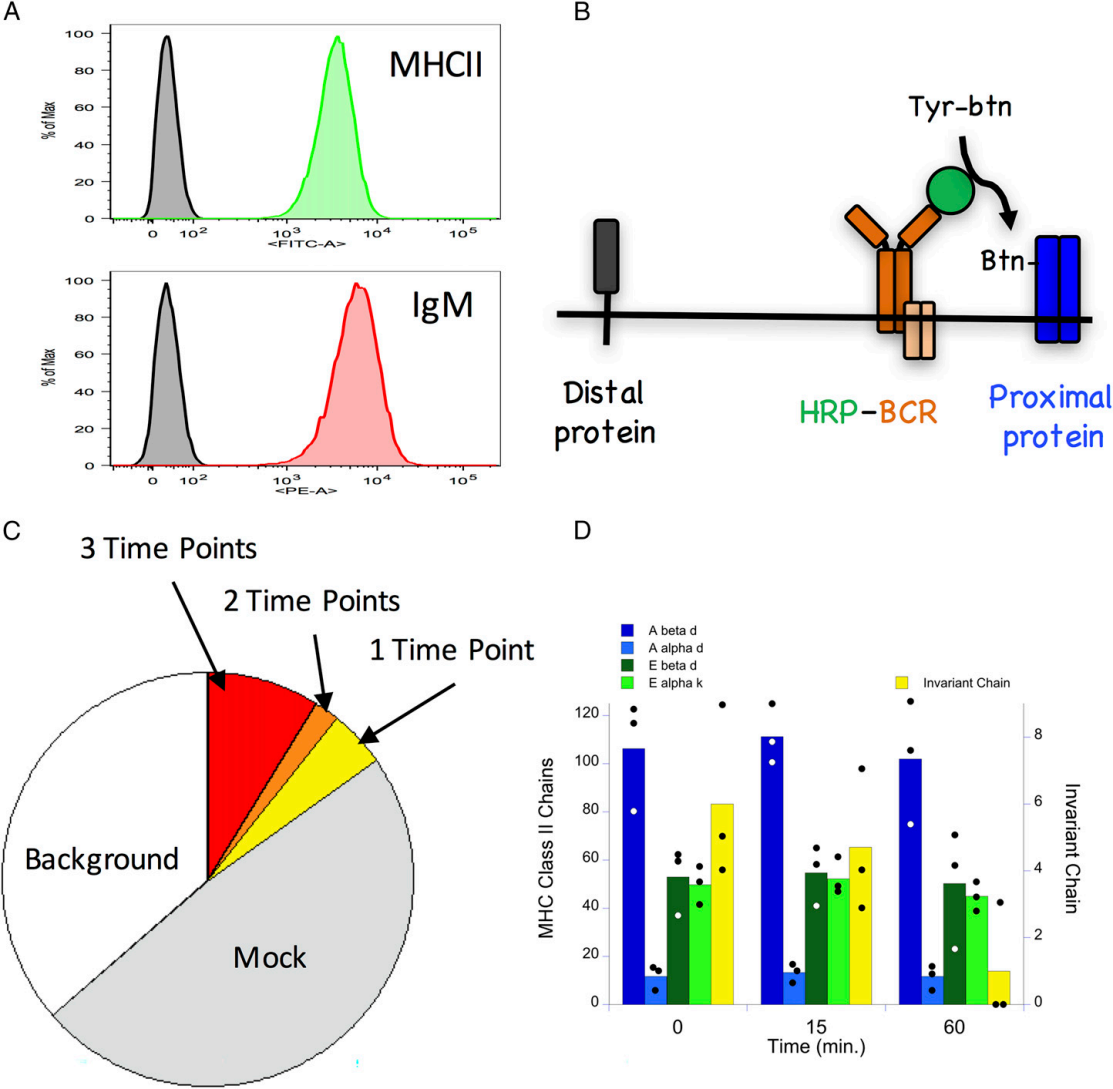
SPPLAT-based analysis of BCR molecular neighbors. (**A**) K46µ B cells were stained for I-A^d^ class II (AMS-32.1-FITC) or IgM BCR (DS-1-PE) and analyzed by flow cytometry. Shown are histograms of events gated on signal cells by FCS/SSC. (**B**) Diagram of BCR SPPLAT approach. Anti–IgM-HRP is bound to cell-surface BCR molecules. The cells are exposed to tyramide-biotin (tyr-btn) and H_2_O_2_. HRP catalyzes selective biotinylation of neighboring proteins. (**C**) Subsequent to BCR-SPPLAT labeling (see Materials and Methods), biotin-tagged molecules were recovered with SA-beads, and the captured proteins were analyzed by MS (see Materials and Methods). The breakdown of the different categories of identified proteins is shown. (**D**) Plotted are the average MS counts across three independent experimental runs for the five listed MHCII chains (points represent values across three independent experiments; see Supplemental Material).

The BCR is a multichain membrane protein with multiple functions, such as signaling and the endocytosis of bound Ag. Neighboring molecules/structures within the membrane can impact on BCR function. For example, membrane lipids (i.e., lipid rafts) and membrane proteins, such as CD20 and FcγRII, can dramatically impact BCR signaling (4–6). To gain a deeper understanding of the various molecular mechanisms controlling BCR function, we used BCR-SPPLAT to biotin-tag BCR molecular neighbors. We then lysed the cells, recovered the biotin-tagged proteins with SA-beads, and identified the isolated proteins by MS. We selected the K46µ B cell line as the primary model for these studies. The K46µ cells express a robust level of both IgM BCR and MHCII molecules (Fig. 1A) and have been used to study many aspects of B cell immunobiology (e.g., 11, 16–18). This approach provides us the opportunity to use BCR-SPPLAT to extend the work of Li et al. (7) and investigate the potential interactions between the IgM BCR and molecules involved in Ag processing/presentation.

Anti–IgM-HRP was bound to cell-surface IgM BCR molecules, and the cells then were incubated at 37°C for 0–60 min to allow redistribution of BCR molecules. BCR molecular neighbors were then labeled via BCR-SPPLAT (Fig. 1B); the cells lysed in detergent and biotin-tagged proteins were harvested with SA-beads. The molecular composition of the samples was determined by MS (see Materials and Methods for details). Samples derived from cells that underwent mock SPPLAT labeling (no H_2_O_2_ or tyramide-biotin) served as a negative control. Because the tyramide-biotin SPPLAT reagent does not readily cross the lipid bilayer, this proximity-based labeling approach will primarily target cell-surface proteins.

Three independent runs of the BCR-SPPLAT/MS experiment identified a total of 679 proteins captured by the SA-beads (Fig. 1C, Supplemental Material). Of these, 577 (85.0%) were considered “background” hits because they were either detected in samples prepared from “mock” SPPLAT-treated cells (330 proteins) or seen in only one or two experiments (247 proteins, most detected at very low levels). Of the remaining 102 proteins detected in all three experimental runs, 58 (56.9%) were detected at all three time points, 14 (13.7%) were detected at two of three time points, and 30 (29.4%) were detected at a single time point (Fig. 1C). Of the 102 proteins detected in all three experiments, there are members from a few notable biological families (see later and Supplemental Material).

### Bioinformatic analysis of the set of SPPLAT-labeled BCR neighbors

The UniProt accession numbers of the 102 BCR neighbors identified by BCR-SPPLAT were uploaded to the National Institutes of Health Database for Annotation, Visualization and Integrated Discovery (DAVID) server (https://david.ncifcrf.gov/) (19). The results of this analysis (limited to *mus musculus*) are summarized in Table I. The CC terms most highly associated with the set of BCR neighbors are “Cell Membrane,” “Membrane,” and “Cell Surface,” consistent with the method of sample preparation because BCR-SPPLAT labeling would be expected to selectively label cell-surface membrane proteins. Further consistent with high selectivity of the labeling method is the enrichment of the posttranslational modifications (PTMs) of “disulfide bonds” and “glycoproteins,” which would be expected for membrane proteins. This indicates that BCR-SPPLAT labeling is selective for the types of protein expected to reside within the BCR’s molecular neighborhood.

**Table I.** Summary of DAVID analysis of BCR-SPPLAT–labeled proteins

DAVID Category	Term	Gene Count	Gene %	*p* Value	Benjamin
CC	Cell membrane	66	60	3.2E−20	8.2E−19
CC	Membrane	95	86.4	2.5E−17	3.2E−16
CC	MHCII	4	3.6	6.7E−5	2.9E−4
MF	Integrin	8	7.3	2.9E−9	1.0E−7
MF	Receptor	26	23.6	3.6E−4	6E−3
PTM	SS-bond	67	60.9	5.9E−15	1.1E−13
PTM	Glycoprotein	71	64.5	2.2E−12	2.1E−11
PTM	Palmitate	17	15.5	8.3E−8	5.3E−7
CC_Direct	Plasma membrane	98	89.1	7.0E−40	1.9E−37
CC_Direct	External side of plasma membrane	42	38.2	9.6E−32	1.3E−29
CC_Direct	Cell surface	38	34.5	1.8E−25	1.6E−23
CC_Direct	Membrane	87	79.1	3.1E−19	2.0E−17
MF_Direct	Protein binding	63	57.3	7.1E−11	2.1E−8
MF_Direct	Integrin binding	11	10.0	1.8E−8	1.9E−6
MF_Direct	MHCII protein complex binding	6	5.5	2.0E−8	1.9E−6

There are two additional CC/molecular function (MF) terms highly associated with the set of BCR neighbors: integrins (discussed later) and MHCII molecules. Direct analysis of the list of SPPLAT-labeled BCR neighbor proteins (Supplemental Material) reveals that three of four MHCII chains (I-Aα, I-Eα, and I-Eβ) are within the top 13 most abundant BCR-SPPLAT–labeled proteins in samples from all three time points, with strong signals seen for all four proteins (α- and β-chains of both I-A and I-E) across all three time points (Fig. 1D, Table II). Class II–associated Ii was also detected in all BCR-SPPLAT samples, albeit at lower levels (Fig. 1D, Table II). Together, these results suggest a robust level of close physical association between Ag–BCR complexes and cell-surface MHCII molecules, including nascent MHCII–Ii complexes.

**Table II.** Summary of BCR-SPPLAT labeling of MHCII molecules

Class II	Accession No.	Exclusive Unique Peptides*^a^*	Coverage, Amino Acids (%)*^a^*
Aα	P04228	7	71/256 (28)
Aβ	P01921	14	106/265 (40)
Eα	P04224	12	84/255 (33)
Eβ	P01915	13	83/264 (31)
Ii	P04441	6	50/279 (18)^*b*^

a Average values calculated from across all three experimental replicates.

b All detected peptides are common to both p31 and p41 isoforms of Ii (i.e., no peptides unique to the p41 Ii isoform were reported).

### MHCII is a persistent BCR neighbor

**FIGURE 2.**
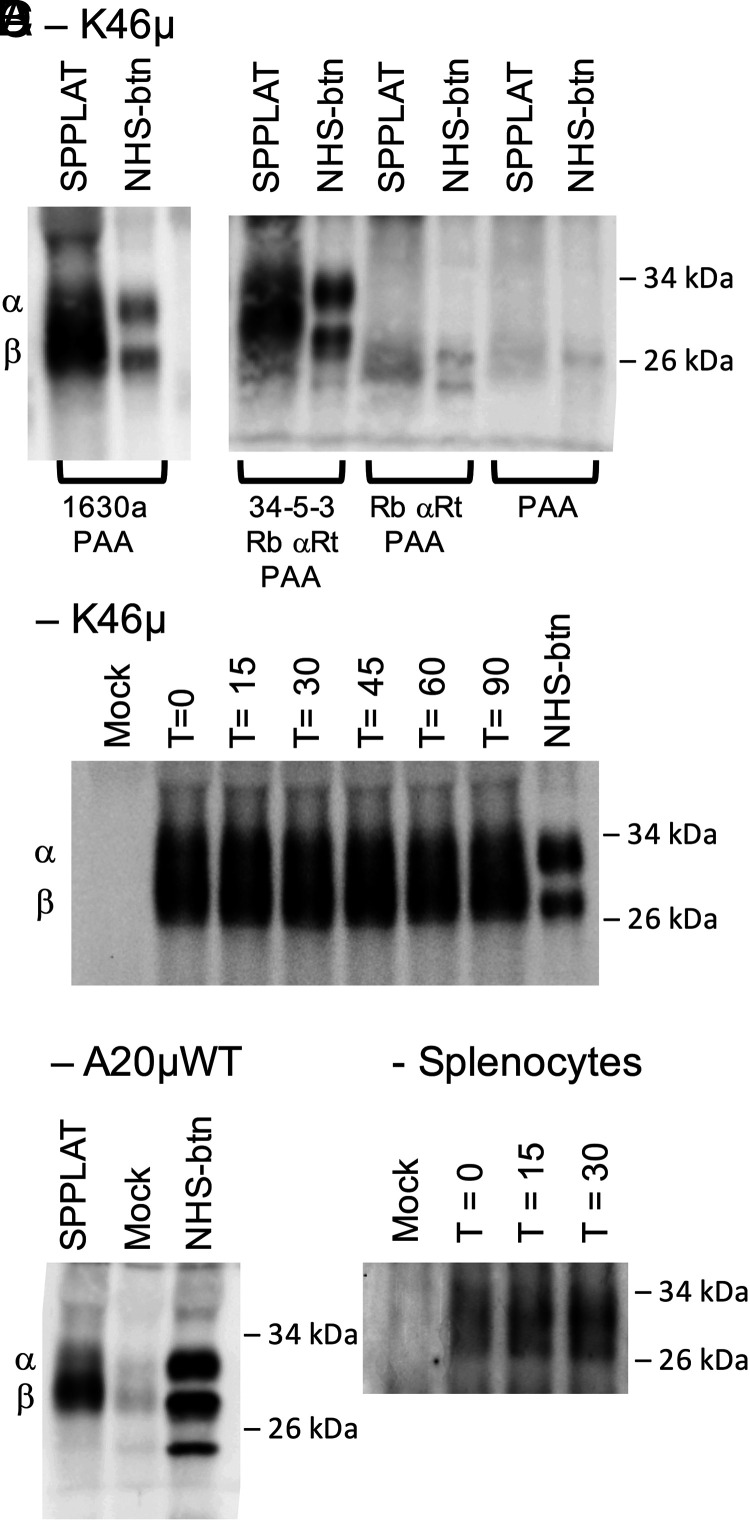
BCR-SPPLAT labeling of MHCII. (**A**) K46µ B cells were labeled by BCR-SPPLAT or NHS-biotin (to label all cell-surface proteins). Lysates from labeled cells were immunoprecipitated with anti-MHCII as indicated and then probed for biotinylated proteins by SA-HRP Western blot. (**B**) Anti–IgM-HRP was bound to K46µ B cells, and the cells were incubated at 37°C for the indicated times (to allow redistribution of cell-surface proteins). The cells were then processed for BCR-SPPLAT labeling and analyzed for biotin-tagged MHCII as in (A) (1630a IP, 1603a is a rabbit Ab against the cytoplasmic tail of the I-A β-chain). (**C**) Anti-human IgM-HRP was bound to A20µWT B cells expressing a transfected human IgM BCR (37). The cells were then processed for BCR-SPPLAT labeling/MHCII IP as in (A). (**D**) Anti-mouse IgM-HRP was bound to splenic B cells. The cells were then processed for BCR-SPPLAT labeling/MHCII IP as in (A). For all panels, representative results from one of three or more independent experiments are shown.

In previous studies, we used coimmunoprecipitation (co-IP) and FRET to reveal a physical association between Ag–BCR complexes and MHCII molecules, primarily within intracellular compartments (11). The earlier results suggest that the association between BCR and MHCII molecules is more pervasive and also occurs at the cell surface. To further investigate this possibility, we again performed BCR-SPPLAT labeling with K46µ cells [the B cell line that was used for the published FRET-based studies (11)], but the resulting samples were analyzed by two different pull-down/Western blot approaches. For the first approach, MHCII molecules were isolated from WCLs with various anti–class II Abs and protein A-beads, and the samples were then probed for biotin-tagged MHCII by SDS-PAGE and blotting with streptavidin-HRP (SA-HRP). This approach led to detection of biotin-tagged class II α- and β-chains (Fig. 2A), confirming the SPPLAT/MS results discussed earlier. Incubation of the anti–IgM-HRP–tagged K46µ B cells at 37°C before running the BCR-SPPLAT reaction reveals that BCR-MHCII proximity can be detected across the entire time course of the experiment (0–90 min; Fig. 2B). Similar results were obtained with the A20µWT B cell line that expresses a transfected human IgM BCR and I-A^d^ class II, and that were used for the previous co-IP analysis of BCR–class II association (Fig. 2C) (11) and also with splenic B cells (Fig. 2D).

**FIGURE 3.**
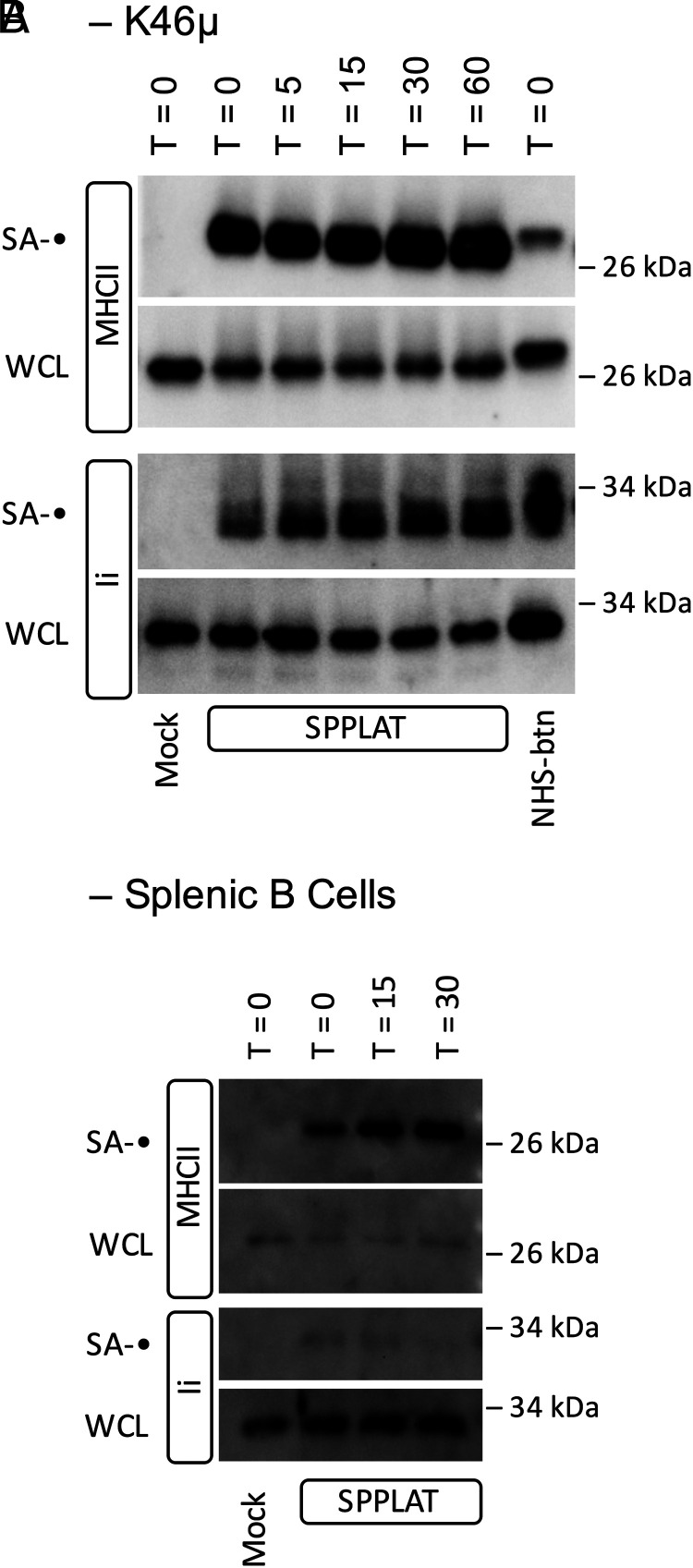
BCR-SPPLAT labeling of MHCII and Ii. (**A**) K46µ B cells were processed for BCR-SPPLAT as detailed in Fig. 2. Biotin-tagged proteins were collected from WCL with SA-beads and probed by Western blot (along with WCL as a control) for MHCII β-chain or Ii. (**B**) Splenic B cells were processed for BCR-SPPLAT labeling as detailed earlier and analyzed for biotin-tagged MHCII β-chain and Ii as in (A). Representative results from one of three or more independent experiments are shown.

For the second approach, biotin-tagged molecules were purified from the WCL of BCR-SPPLAT–labeled cells using SA-beads, and the samples were then probed for MHCII by SDS-PAGE and Western blotting with an anti–class II β-chain Ab. This approach led to detection of SPPLAT-labeled MHCII across the entire time course of the experiment for both K46µ B cells (Fig. 3A) and splenic B cells (Fig. 3B). One advantage of this second approach is that Western blot analysis of the SA-isolated BCR-SPPLAT–labeled proteins with additional Abs can lead to the detection of additional BCR molecular neighbors. Accordingly, we probed the SA pull-downs with an anti-Ii Ab. Ii (CD74) is an MHCII chaperone that aids class II folding in the endoplasmic reticulum and shepherds nascent class II molecules to intracellular compartments for peptide loading. This additional blotting led to reproducible detection of BCR-SPPLAT–labeled Ii for K46µ B cells (Fig. 3A) and splenic B cells (Fig. 3B). These results confirm the detection of BCR-SPPLAT–labeled Ii by MS (see earlier), suggesting that at least a portion of the BCR-associated MHCII are nascent molecules, primed for peptide loading.

**FIGURE 4.**
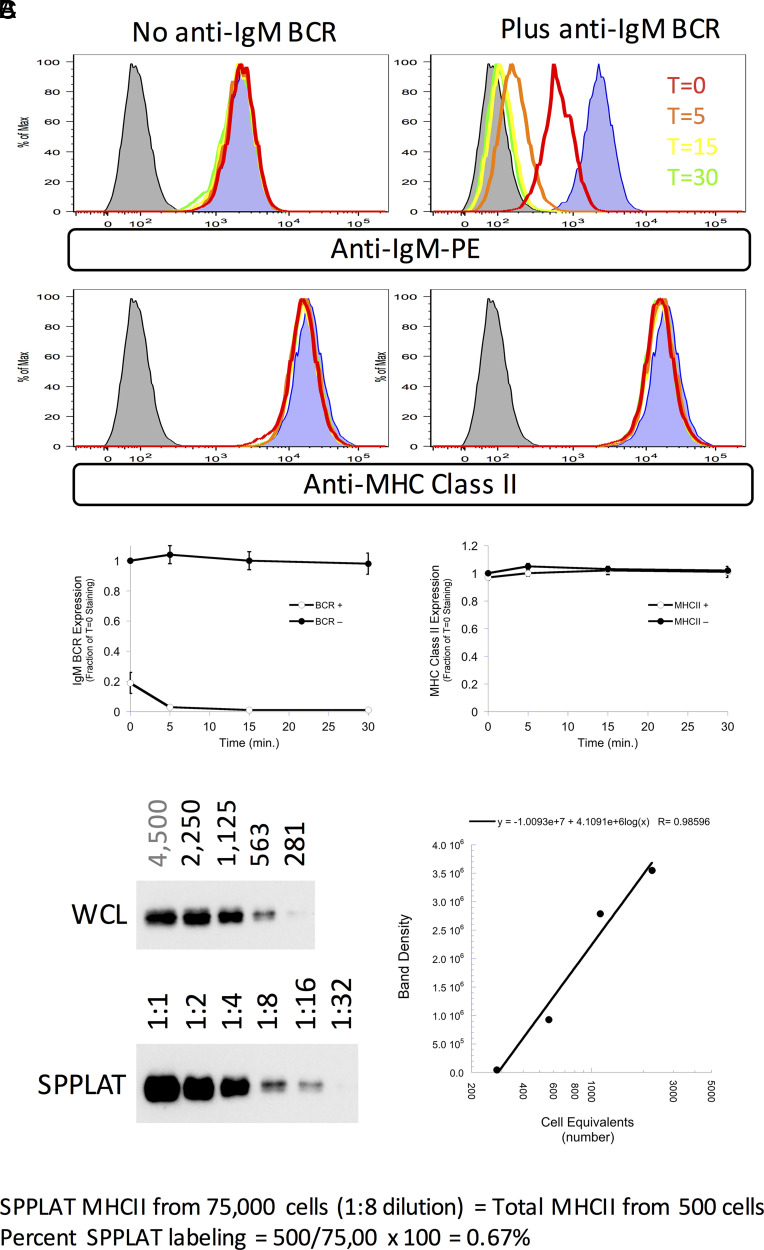
A small fraction of MHCII associates with the BCR. (**A**) K46µ B cells were untreated or treated with anti-IgM Ab to induce BCR cross-linking and downregulation. The cells were then stained for cell-surface BCR and MHCII molecules and analyzed by flow cytometry. Histograms of staining gated on single cells by FCS/SSC are shown. (**B**) Quantitation of BCR and MHCII cell-surface expression averaged across three independent experiments. Error bars indicate ±1 SD. (**C**) WCL from known numbers of K46µ B cells was subject to SDS-PAGE/Western blot for MHCII β-chain. Densitometry of the resulting blot produced a standard curve. Serial dilution of SA pull-down from BCR-SPPLAT–labeled cells (BCR-proximal MHCII) was also probed for MHCII β-chain. The signal for the SA pull-down was compared with the standard curve. Calculation of the fraction of BCR-SPPLAT–labeled MHCII is shown. Results from one of three independent experiments are shown.

One outstanding question raised by the earlier results is: “What fraction of MHCII molecules is found in close proximity to the BCR?” If a large fraction of cell-surface MHCII is BCR associated, then downregulation of cell-surface BCR could result in the codownregulation of a detectable amount of cell-surface MHCII. However, experiments designed to test this idea did not reveal any detectable level of MHCII downregulation in B cells when BCR internalization was triggered (Fig. 4A, 4B). Although this result might seem in contradiction to the results of Hernández-Pérez et al. (20), who report colocalization of coincident internalized Ag–BCR complexes and MHCII in early endocytic compartments, in that report class II molecules were “prelabeled” (tagged with fluorescently labeled anti–class II Ab), which could independently drive MHCII cross-linking and internalization into BCR-containing endosomes.

To directly quantify the level of BCR-SPPLAT–labeled MHCII, we generated serial dilutions of both WCL and BCR-SPPLAT/SA pull-down and determined the relative level of class II in each by Western blot (Fig. 4C). Head-to-head comparison of the class II levels in the two samples reveals that BCR-bound anti–IgM-HRP can SPPLAT-label ∼1% (average of 0.74% across three independent experiments) of total cellular MHCII molecules. Although this may seem like a small fraction of MHCII molecules, at least some of these MHCII molecules are Ii associated (nascent), and this highly selected MHCII subset may have unique immunological functions (see Discussion).

### The tetraspanin protein CD37 as a BCR neighbor and modulator of B cell function

SPPLAT labeling of BCR molecular neighbors led to identification of a family of proteins involved in the formation of membrane microdomains, including CD37, raftlin, and Ig superfamily member 8 (IgSF8, also known as EWI-2; Table III). Raftlin, a lipid raft protein, was initially identified in chicken B cells and was shown to be involved in BCR signaling (21). In human B cells, IgSF8/EWI-2 was shown by co-IP to associate with the tetraspanin CD9 (22, 23). In the same studies, CD9 was also shown to associate with HLA-DR and IgM, but it is unclear whether IgSF8/EWI-2 is associated with the same CD9 molecules as HLA-DR and IgM. Here, it should be noted that tetraspanin microdomains and lipid rafts represent two distinct types of microdomain (24). Thus, BCR-SPPLAT labeling of components of each type of microdomain suggests that BCR molecules can access both compartments.

**Table III.** Summary of BCR-SPPLAT labeling of membrane domain proteins

Protein	Accession No.	Exclusive Unique Peptides^*a*^	Coverage, Amino Acids (%)^*a*^
CD37	Q61470	6	49/281 (17)
Raftlin	Q6A0D4	4	56/554 (10)
IgSF8	Q8R366	11	112/611 (18)

a Average coverage across nine samples (three time points across three independent experiments).

Although CD37 has *not* previously been identified as a BCR-associated protein, CD37Δ mice have been shown to have defects in T cell–dependent humoral immune responses (25), responses that require efficient BCR-mediated Ag processing/presentation. CD37 has also been reported to have both an ITAM and an ITIM motif (26), suggesting that it might impact BCR signaling. Finally, CD37 was shown to associate with MHCII molecules and MHCII signaling molecules, such as SCIMP (27). Therefore, we decided to investigate any potential role of CD37 in BCR function.

**FIGURE 5.**
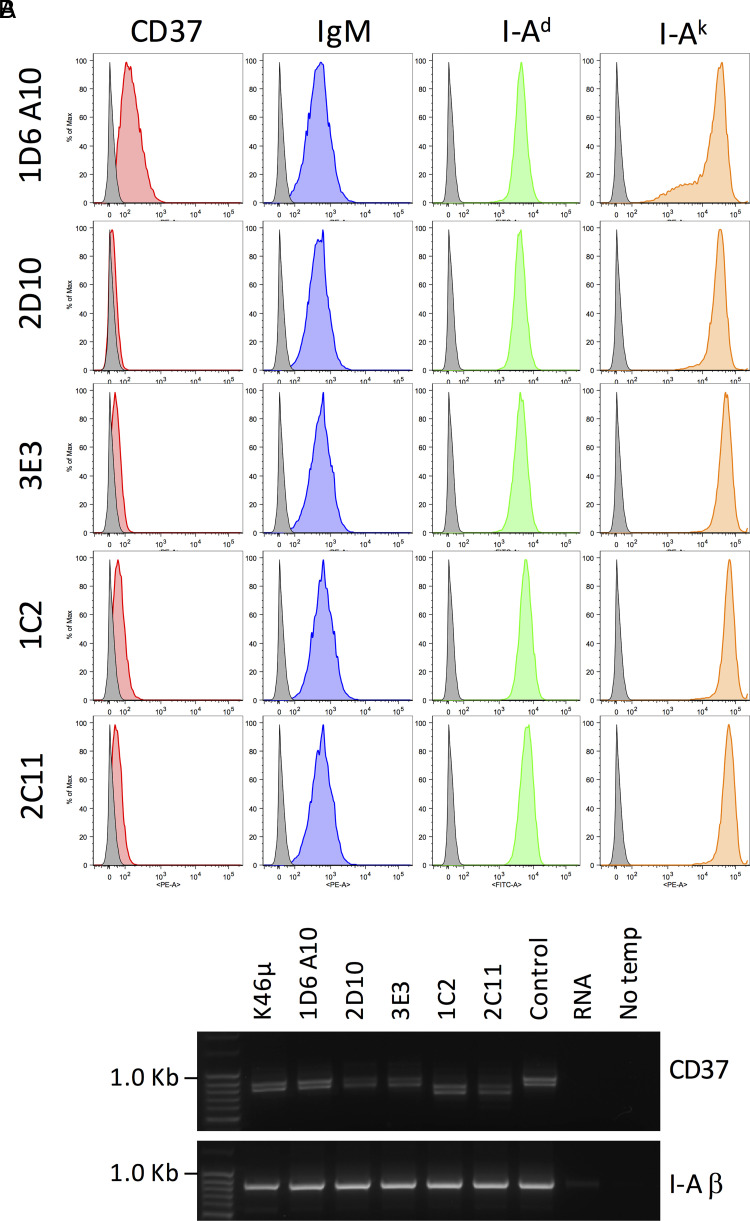
Generation and characterization of CD37Δ 1D6 A10 B cells. (**A**) 1D6 A10 B cells (K46µ B cells expressing transfected I-A^k^ MHCII) (11) were transfected with CRISPR vectors to ablate CD37 expression (see Materials and Methods). Drug-selected cells were cloned by limiting dilution, selected clones stained for CD37 (Duno85-PE), IgM BCR (DS-1-PE), endogenous I-A^d^ class II (AMS-32.1-FITC), and transfected I-A^k^ class II (10-3.6-PE), and the cells were analyzed by flow cytometry. Histograms of staining gated on single cells by FCS/SSC are shown. (**B**) PCR analysis of cDNA from the indicated cells using primers covering >90% of the CD37 coding region (849/912 bp, including the start codon; see Materials and Methods). Control samples are from cells transfected with the nontargeting CRISPR plasmid (Control) or RNA or no PCR template (No temp). PCR analysis of the I-A^d^ β-chain was used as a control. The 2C11 clone exhibits unusual morphology and growth kinetics, and thus was excluded from further analysis.

Two primary BCR functions triggered by Ag binding are ITAM-based signaling and endocytosis of Ag–BCR complexes, functions that involve membrane domains such as “lipid rafts” and clathrin-coated pits (2, 28). To probe the potential role of the tetraspanin CD37 in these two BCR functions, we used a CRISPR-based approach to generate CD37 knockout (CD37Δ) B cells. Here, we used K46µ B cells expressing a transfected I-A^k^ MHCII molecule (i.e., 1D6 A10 cells) (11). We obtained >100 drug-resistant clones, screened 47 for CD37 expression by flow cytometry, and selected 4 for further analysis. Although the parental B cell line expresses readily detectable levels of cell-surface CD37, there is little, if any, CD37 staining of the four selected CD37Δ clones (Fig. 5A). Ablation of CD37 expression does not appreciably alter the expression of the BCR or MHCII molecules (Fig. 5A). RT-PCR analysis of the four clones revealed no change in CD37 mRNA size for the 2D10 and 3E3 clones (generated with the TEVM-1129421 plasmid, suggesting a point mutation) or a decrease in CD37 mRNA size for the 1C2 and 2C11 clones (generated with the TEVM-11966563 plasmid, suggesting a deletion).

**FIGURE 6.**
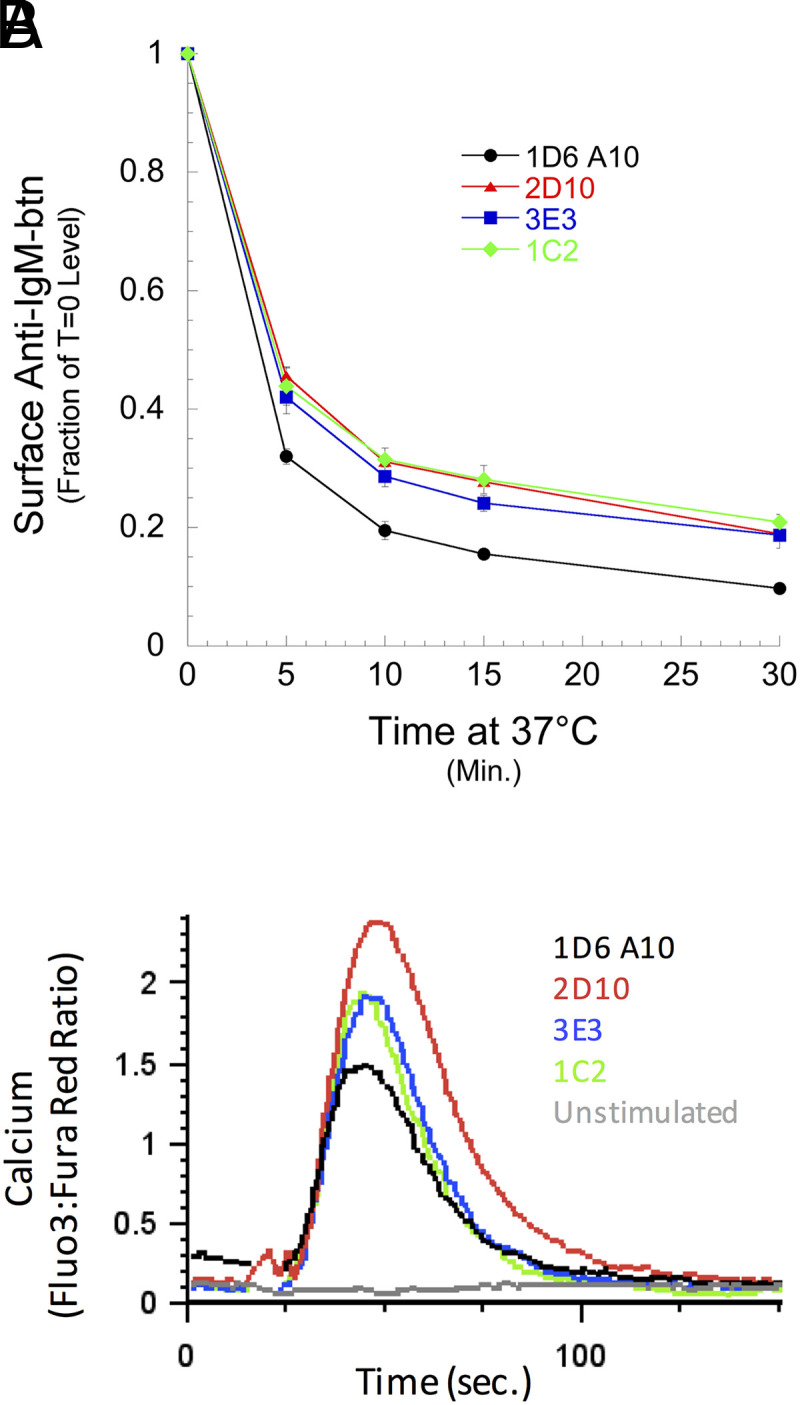
Ablation of CD37 expression alters BCR endocytosis and signaling. (**A**) Anti–IgM^a^-btn was bound to 1D6 A10 B cells and CD37Δ derivatives, and the cells were incubated at 37°C for the indicated times. Remaining cell-surface anti–IgM^a^-btn was detected with SA-PE and the cell analyzed by flow cytometry. Mean fluorescence intensity values were normalized to T = 0 binding for each cell line. Error bars indicated ±1 SD across three independent experiments. Comparison of the final level of clearance by a Student *t* test (wild-type versus each CD37Δ line) returned a *p* value < 0.005 for each mutant. (**B**) 1D6 A10 B cells and CD37Δ derivatives were loaded with the Fluo-3 and Fura Red calcium-sensitive dyes. Baseline fluorescence was determined for 15 s by flow cytometry, and the cells were then stimulated with 3 µg/ml NP-HEL and fluorescence monitored for a total of 5 min. The ratio of Fluo-3 to Fura Red signal is reported as a readout of intracellular calcium levels. Representative results from one of three independent experiments are shown.

To investigate the potential effects of CD37 on BCR endocytosis and signaling, we determined the kinetics of BCR endocytosis and calcium signaling in CD37Δ B cells. To analyze BCR endocytosis, we determined the kinetics of endocytosis of BCR-bound anti-IgM Ab in CD37Δ and parental B cells (Fig. 6A). The results reveal that cells lacking CD37 exhibit slowed kinetics of BCR internalization. To investigate BCR signaling, we followed the kinetics of Ag-triggered changes in intracellular calcium (Fig. 6B). These results reveal that CD37Δ B cells exhibit elevated levels of BCR-triggered intracellular calcium signaling. Thus, the BCR molecular neighbor and membrane microdomain protein CD37 is able to modulate the levels of both BCR signaling and BCR-mediated Ag endocytosis.

**FIGURE 7.**
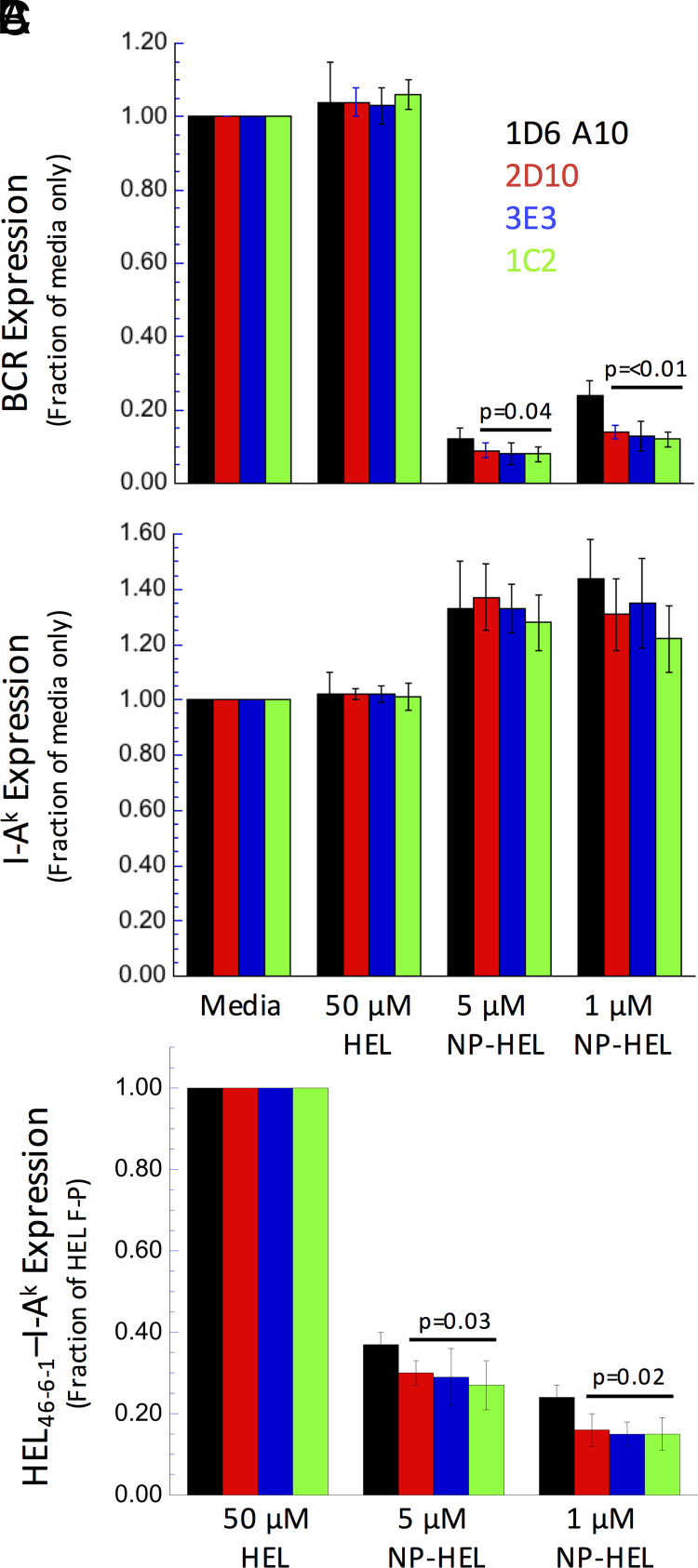
CD37 knockout tempers BCR-mediated formation of peptide–MHCII complexes. 1D6 A10 B cells and CD37Δ derivatives were pulsed overnight with the indicated doses of either HEL (fluid-phase Ag processing, a control) or NP-HEL (BCR-mediated Ag processing). The cells were harvested and stained for IgM^a^ BCR (DS-1-PE) (**A**), I-A^k^ class II (11-5.2-FITC) (**B**), or, in a second tube, HEL_46-61_–I-A^k^ complexes (C4H3-FITC) (**C**). Staining for the IgM BCR and class II is normalized to the staining observed with cells incubated in media only. Staining for HEL_46-61_–I-A^k^ complexes is normalized to the staining observed with cells pulsed with 50 µM HEL. Average values across three independent experiments are shown. Error bars indicate ±1 SD. The reported *p* values are derived by comparing results from all three CD37Δ cells as a group with results from the parental cells (there are no statistical differences in the level of MHCII upregulation).

The ultimate fate of BCR-internalized cognate Ag is conversion to peptide–MHCII complexes, which are then expressed by the B cell to recruit T cell help. To investigate the potential role of CD37 in this final phase of BCR function, we determined the level of peptide–class II complexes formed by parental and CD37Δ cells. All cells express roughly similar levels of I-A^k^ MHCII molecules and an NP-specific IgM BCR (Fig. 5A). The cells were pulsed overnight under four different conditions: media only (negative control), 50 µM HEL (fluid-phase Ag processing that bypasses the BCR; positive control), 5 µM NP-HEL, or 1 µM NP-HEL (two Ag doses for BCR-mediated processing and presentation). After overnight culture, B cells pulsed with media or HEL express high levels of IgM BCR, whereas cells pulsed with either dose of NP-HEL exhibit robust BCR downregulation, indicating recognition and endocytosis of NP-HEL (Fig. 7A). For *total* cell-surface I-A^k^ class II, cells cultured in media or 50 µM HEL exhibit similar levels of class II expression, whereas cells pulsed with NP-HEL (which triggers BCR signaling; Fig. 6B) exhibit an ∼30% increase in class II expression (Fig. 7B). Turning to specific HEL_46–61_–I-A^k^ complexes, in each experiment we set the level of such complexes [detected by binding of the C4H3 mAb (12, 15)] in cells pulsed with 50 µM HEL (fluid-phase Ag processing) to 1.00 and determined the level of complexes formed from NP-HEL (BCR-mediated Ag processing) in relation to this benchmark (see Materials and Methods for details). Although the impact of CD37 ablation is not complete, the three NP-HEL–pulsed CD37Δ cell lines tested express statistically significantly lower levels of peptide–class II complexes compared with NP-HEL–pulsed parental cells (Fig. 7C). Because we are focused on determining the impact of CD37 deletion on B cell–intrinsic aspects of Ag processing/presentation, we elected to use C4H3 mAb binding over T cell activation as a readout of the levels of peptide–class II expression because T cell activation can be impacted by additional variables such as partitioning of peptide–class II complexes into membrane microdomains (29). This finding is in line with the observation that CD37Δ mice exhibit an impaired T cell–dependent humoral immune response *under conditions of limited costimulation* (discussed later). Because tetraspanin proteins can sometimes have overlapping functions (30), it would be interesting in future work to determine whether double knockout of CD37 and additional BCR-proximal tetraspanins, such as IgSF8 (Table III), would result in a more pronounced effect (Table IV).

**Table IV.** Comparison of DT-40 versus K46µ SPPLAT results

Protein	DT-40	K46µ
Na-K ATPase	α1β3	α1β3
Integrin	α3β1	α6, αL, αV, α4 / β1, β2, β3
Ig μ-chain C region	√	√
CD79b (little/no labeling of CD79a)	√	√
PTPRC/CD45	√	√
chB6	√	—
MHCII	—	√
Raftlin	√	√
CD37^*a*^	—	√
CD20 (atypical tetraspanin)	—	√
IgSF8/CD316/PGRL/EWI-2^*b*^	—	√

Dashes indicate a lack of detection.

a Tetraspanin family proteins.

b Tetraspanin interacting proteins (interacts with CD9 and CD81) (45).

## Discussion

The BCR drives Ag-specific clonal selection of B cells, the process that underlies the high level of Ag specificity of the humoral immune response. Ag binding to the BCR drives signaling and Ag endocytosis/processing/presentation, leading to B cell activation and recruitment of T cell help. Proteins and lipids neighboring the BCR can impact BCR function and thus impact the immune response. To gain a deeper understanding of the set of BCR-proximal molecules that may modulate BCR function, we use a proximity-based biotinylation technique, paired with identification of tagged proteins by SA pull-down/MS (i.e., BCR-SPPLAT). The BCR-SPPLAT approach tagged ∼100 BCR neighbor proteins, including MHCII molecules, integrins, transporters, and membrane microdomain components. Moreover, ablation of expression of one BCR-proximal membrane microdomain protein, CD37, alters the dynamics of BCR signaling, endocytosis, and Ag processing. In future studies, it will be interesting to look at the specific impact of CD37 on the precise trafficking of Ag–BCR complexes through the endocytic pathway, multiple additional aspects of BCR signaling, and the molecular mechanism of formation of peptide–class II complexes.

Membrane microdomains such as lipid rafts and clathrin-coated pits are central to BCR function (2, 28). Raftlin is a lipid raft protein originally identified in B cells and is known to play an important role in BCR signaling (21). BCR-SPPLAT labeling studies with both chicken DT-40 B cells (7) and murine K46µ B cells (this study) tagged raftlin as a BCR molecular neighbor (Table IV). This observation establishes the ability of BCR-SPPLAT to tag BCR-proximal membrane microdomain proteins critical for BCR function.

The studies presented in this report are an extension of the original studies of Li et al. (7), who used the BCR-SPPLAT approach to define the IgM BCR molecular neighbors on chicken DT-40 B cells. Although there are many similarities between the results of the two studies (Table IV and discussed later), this study extends the analysis to MHCII-expressing B cells and reveals that class II molecules are in close physical proximity to cell-surface BCR molecules. These findings are in line with our previous studies where we used FRET and co-IP to reveal an association of the BCR and MHCII molecules primarily within intracellular compartments (11). As discussed in that report, close proximity of MHCII molecules with Ag–BCR complexes suggests that peptides derived from the processing of BCR-bound cognate Ag end up selectively loaded onto BCR-associated class II molecules. Consistent with that notion is the current observation that SPPLAT labeling tags both class II α- and β-chains, as well as class II–associated Ii, indicating that at least a subset of BCR-associated MHCII are nascent molecules ready to be converted to peptide–MHCII complexes. This observation is also in line with the observations by Hauser and Lindner (31) that BCR cross-linking results in coalescence of the BCR and class II–Ii complexes in lipid rafts. Interestingly, in addition to being a key component of the BCR, CD79 is known to associate with MHCII molecules (17, 18). Moreover, the region of CD79 involved in class II association (i.e., the CD79A connecting peptide) (17) is different from the region of CD79 mediating interaction with the BCR IgH/L core (i.e., the CD79 transmembrane domains) (32–37). This suggests that CD79 may have the ability to simultaneously interact with the Ig core of the BCR *and* MHCII molecules, facilitating BCR–MHCII proximity.

Tetraspanins are a family of membrane proteins that can assemble into specialized membrane microdomains (tetraspanin domains) (38). In this study, BCR-SPPLAT labeling tagged three tetraspanins/tetraspanin-interacting proteins as BCR neighbors: CD37, CD20, and IgSF8 (Table IV). For this study, CD37 was particularly interesting because this tetraspanin protein is most highly expressed on B cells and has been reported to possess both an ITAM and an ITIM motif (26). In addition, CD37Δ mice have been reported to exhibit an impaired humoral immune response under conditions of suboptimal costimulation, suggesting a role for CD37 in B cell–T cell interactions (25). Analysis of CD37Δ B cells reveals that ablation of CD37 expression results in augmentation of BCR signaling and blunting of BCR-mediated Ag endocytosis/processing/presentation (Figs. 6, 7). This inverse relationship between BCR signaling and endocytosis is consistent with previous observations that BCR signaling-associated CD79 ITAM phosphorylation results in the inactivation of ITAM-embedded BCR endocytosis motifs (2, 3) and could underlie the deficit in T cell–based humoral immunity observed in CD37Δ mice (25). Intriguingly, CD37 is also known to interact with MHCII (39), as well as with SCIMP (a transmembrane adaptor protein involved in MHCII signaling) (27), suggesting that CD37 might be directly or indirectly involved in the earlier-detailed association between the BCR and MHCII molecules. These potential interactions are worthy of additional study.

Previous studies have shown a functional association between the BCR and integrins in that BCR cross-linking drives integrin activation (40). In DT-40 B cells, Li et al. (7) show by BCR-SPPLAT labeling that α3β1 integrin is a molecular neighbor of the BCR. They also demonstrated that Ab engagement of either the BCR or an additional BCR neighbor chB6 (an Ig-domain–bearing membrane protein) stimulates integrin-mediated B cell adhesion to laminin-coated plates (7). In this study, we found multiple BCR-SPPLAT–labeled integrin subunits (Table IV). Although we have not probed any potential functional links between the BCR and integrins, each of the BCR-SPPLAT–labeled α or β integrin subunits that we detected is capable of heterodimerizing with another subunit among those detected to form a functional integrin molecule (i.e., α6β1, αLβ2, αVβ3, and α4β1) (41). Interestingly, the tetraspanin protein CD37 (see earlier) has been shown to orchestrate α4β1-Akt signaling to support long-lived plasma cell survival (42). Together, these findings suggest a physical and a functional link between the BCR, integrins, and other BCR molecular neighbors, interactions that may be important to B cell immunobiology.

In both this study and that of Li et al. (7), the α1 and β3 subunits of the plasma membrane Na/K ATPase were robustly BCR-SPPLAT tagged (Table IV). Although the potential implications of the close physical proximity of the BCR to this key membrane transporter are not immediately obvious, it has been previously reported that a mAb against the Na/K ATPase β3 subunit blocks anti-IgM–induced proliferation of human peripheral blood B cells (43). This functional and physical link between the BCR and Na/K ATPase is worthy of further investigation.

In summary, the use of BCR-SPPLAT to label/identify BCR-proximal proteins led to the identification of ∼100 molecular neighbors of the BCR, which fall into multiple functional groups, including MHCII molecules and membrane microdomain proteins. The proximity of the BCR and nascent MHCII molecules is consistent with findings from previous studies (11, 31) and suggest a coordinated mechanism for the highly controlled loading of cognate Ag-derived peptides onto specific subsets of MHCII molecules (11, 12, 44). The proximity of the BCR to tetraspanin proteins such as CD37 and the impact of CD37 knockout on BCR function indicate a role of tetraspanin proteins and tetraspanin membrane microdomains in BCR function. Future analysis of the other BCR molecular neighbors holds the potential to uncover additional unappreciated modulators of BCR function.

## Supplementary Material

Supplemental Material_1 (XLS)

Supplemental Material_2 (PDF)

## References

[1] Tkachenko, A., K. Kupcova, O. Havranek. 2024. B-cell receptor signaling and beyond: the role of Igα (CD79a)/Igβ (CD79b) in normal and malignant B cells. Int. J. Mol. Sci. 25: 10. 10.3390/ijms25010010 PMC10779339 38203179

[2] Busman-Sahay, K., L. Drake, A. Sitaram, M. Marks, J. R. Drake. 2013. Cis and trans regulatory mechanisms control AP2-mediated B cell receptor endocytosis via select tyrosine-based motifs. PLoS One 8: e54938. 23372794 10.1371/journal.pone.0054938 PMC3553015

[3] Hou, P., E. Araujo, T. Zhao, M. Zhang, D. Massenburg, M. Veselits, C. Doyle, A. R. Dinner, M. R. Clark. 2006. B cell antigen receptor signaling and internalization are mutually exclusive events. PLoS Biol. 4: e200. 16719564 10.1371/journal.pbio.0040200 PMC1470458

[4] Getahun, A., J. C. Cambier. 2015. Of ITIMs, ITAMs, and ITAMis: revisiting immunoglobulin Fc receptor signaling. Immunol. Rev. 268: 66–73. 26497513 10.1111/imr.12336 PMC4621791

[5] Petrie, R. J., J. P. Deans. 2002. Colocalization of the B cell receptor and CD20 followed by activation-dependent dissociation in distinct lipid rafts. J. Immunol. 169: 2886–2891. 12218101 10.4049/jimmunol.169.6.2886

[6] Polyak, M. J., H. Li, N. Shariat, J. P. Deans. 2008. CD20 homo-oligomers physically associate with the B cell antigen receptor. Dissociation upon receptor engagement and recruitment of phosphoproteins and calmodulin-binding proteins. J. Biol. Chem. 283: 18545–18552. 18474602 10.1074/jbc.M800784200

[7] Li, X. W., J. S. Rees, P. Xue, H. Zhang, S. W. Hamaia, B. Sanderson, P. E. Funk, R. W. Farndale, K. S. Lilley, S. Perrett, A. P. Jackson. 2014. New insights into the DT40 B cell receptor cluster using a proteomic proximity labeling assay. J. Biol. Chem. 289: 14434–14447. 24706754 10.1074/jbc.M113.529578 PMC4031500

[8] Awoniyi, L. O., D. M. Cunha, A. V. Sarapulov, S. Hernandez-Perez, M. Runsala, B. Tejeda-Gonzalez, V. Sustar, M. O. Balci, P. Petrov, P. K. Mattila. 2023. B cell receptor-induced protein dynamics and the emerging role of SUMOylation revealed by proximity proteomics. J. Cell Sci. 136: jcs261119. 37417469 10.1242/jcs.261119 PMC10445728

[9] Margiotta, A., D. M. Frei, I. H. Sendstad, L. Janssen, J. Neefjes, O. Bakke. 2020. Invariant chain regulates endosomal fusion and maturation through an interaction with the SNARE Vti1b. J. Cell Sci. 133: jcs244624. 32907852 10.1242/jcs.244624

[10] Music, A., B. Tejeda-González, D. M. Cunha, G. Fischer von Mollard, S. Hernández-Pérez, P. K. Mattila. 2022. The SNARE protein Vti1b is recruited to the sites of BCR activation but is redundant for antigen internalisation, processing and presentation. Front. Cell. Dev. Biol. 10: 987148. 36111340 10.3389/fcell.2022.987148 PMC9468668

[11] Barroso, M., H. Tucker, L. Drake, K. Nichol, J. R. Drake. 2015. Antigen-B cell receptor complexes associate with intracellular major histocompatibility complex (MHC) class II molecules. J. Biol. Chem. 290: 27101–27112. 26400081 10.1074/jbc.M115.649582 PMC4646406

[12] Nashar, T. O., J. R. Drake. 2005. The pathway of antigen uptake and processing dictates MHC class II-mediated B cell survival and activation. J. Immunol. 174: 1306–1316. 15661887 10.4049/jimmunol.174.3.1306

[13] Wilson, J. E., B. Katkere, J. R. Drake. 2009. *Francisella tularensis* induces ubiquitin-dependent major histocompatibility complex class II degradation in activated macrophages. Infect. Immun. 77: 4953–4965. 19703975 10.1128/IAI.00844-09 PMC2772548

[14] Drake, J. R. 2019. Signaling cross-talk between MHC class II molecular conformers in resting murine B cells. Immunohorizons 3: 28–36. 31356174 10.4049/immunohorizons.1800078

[15] Zhong, G., C. Reis e Sousa, R. N. Germain. 1997. Production, specificity, and functionality of monoclonal antibodies to specific peptide-major histocompatibility complex class II complexes formed by processing of exogenous protein. Proc. Natl Acad. Sci. USA 94: 13856–13861. 9391117 10.1073/pnas.94.25.13856 PMC28397

[16] Bobbitt, K. R., L. B. Justement. 2000. Regulation of MHC class II signal transduction by the B cell coreceptors CD19 and CD22. J. Immunol. 165: 5588–5596. 11067914 10.4049/jimmunol.165.10.5588

[17] Jin, L., J. C. Stolpa, R. M. Young, A. E. Pugh-Bernard, Y. Refaeli, J. C. Cambier. 2008. MHC class II structural requirements for the association with Igalpha/beta, and signaling of calcium mobilization and cell death. Immunol. Lett. 116: 184–194. 18194817 10.1016/j.imlet.2007.11.023 PMC2424217

[18] Lang, P., J. C. Stolpa, B. A. Freiberg, F. Crawford, J. Kappler, A. Kupfer, J. C. Cambier. 2001. TCR-induced transmembrane signaling by peptide/MHC class II via associated Ig-alpha/beta dimers. Science 291: 1537–1540. 11222857 10.1126/science.291.5508.1537

[19] Sherman, B. T., M. Hao, J. Qiu, X. Jiao, M. W. Baseler, H. C. Lane, T. Imamichi, W. Chang. 2022. DAVID: a web server for functional enrichment analysis and functional annotation of gene lists (2021 update). Nucleic Acids Res. 50: W216–W221. 35325185 10.1093/nar/gkac194 PMC9252805

[20] Hernández-Pérez, S., M. Vainio, E. Kuokkanen, V. Sustar, P. Petrov, S. Forstén, V. Paavola, J. Rajala, L. O. Awoniyi, A. V. Sarapulov, 2019. B cells rapidly target antigen and surface-derived MHCII into peripheral degradative compartments. J. Cell Sci. 133: jcs235192. 31780582 10.1242/jcs.235192

[21] Saeki, K., Y. Miura, D. Aki, T. Kurosaki, A. Yoshimura. 2003. The B cell-specific major raft protein, Raftlin, is necessary for the integrity of lipid raft and BCR signal transduction. EMBO J 22: 3015–3026. 12805216 10.1093/emboj/cdg293 PMC162145

[22] Le Naour, F., S. Charrin, V. Labas, J. P. Le Caer, C. Boucheix, E. Rubinstein. 2004. Tetraspanins connect several types of Ig proteins: IgM is a novel component of the tetraspanin web on B-lymphoid cells. Cancer Immunol. Immunother. 53: 148–152. 14730399 10.1007/s00262-003-0477-5 PMC11032799

[23] Charrin, S., F. Le Naour, V. Labas, M. Billard, J. P. Le Caer, J. F. Emile, M. A. Petit, C. Boucheix, E. Rubinstein. 2003. EWI-2 is a new component of the tetraspanin web in hepatocytes and lymphoid cells. Biochem. J. 373: 409–421. 12708969 10.1042/BJ20030343 PMC1223506

[24] Le Naour, F., M. André, C. Boucheix, E. Rubinstein. 2006. Membrane microdomains and proteomics: lessons from tetraspanin microdomains and comparison with lipid rafts. Proteomics 6: 6447–6454. 17109380 10.1002/pmic.200600282

[25] Knobeloch, K. P., M. D. Wright, A. F. Ochsenbein, O. Liesenfeld, J. Löhler, R. M. Zinkernagel, I. Horak, Z. Orinska. 2000. Targeted inactivation of the tetraspanin CD37 impairs T-cell-dependent B-cell response under suboptimal costimulatory conditions. Mol. Cell. Biol. 20: 5363–5369. 10891477 10.1128/mcb.20.15.5363-5369.2000 PMC85988

[26] Lapalombella, R., Y. Y. Yeh, L. Wang, A. Ramanunni, S. Rafiq, S. Jha, J. Staubli, D. M. Lucas, R. Mani, S. E. M. Herman, 2012. Tetraspanin CD37 directly mediates transduction of survival and apoptotic signals. Cancer Cell 21: 694–708. 22624718 10.1016/j.ccr.2012.03.040 PMC3360882

[27] Draber, P., I. Vonkova, O. Stepanek, M. Hrdinka, M. Kucova, T. Skopcova, P. Otahal, P. Angelisova, V. Horejsi, M. Yeung, 2011. SCIMP, a transmembrane adaptor protein involved in major histocompatibility complex class II signaling. Mol. Cell. Biol. 31: 4550–4562. 21930792 10.1128/MCB.05817-11 PMC3209250

[28] Guo, B., R. M. Kato, M. Garcia-Lloret, M. I. Wahl, D. J. Rawlings. 2000. Engagement of the human pre-B cell receptor generates a lipid raft-dependent calcium signaling complex. Immunity 13: 243–253. 10981967 10.1016/s1074-7613(00)00024-8

[29] Anderson, H. A., E. M. Hiltbold, P. A. Roche. 2000. Concentration of MHC class II molecules in lipid rafts facilitates antigen presentation. Nat. Immunol. 1: 156–162. 11248809 10.1038/77842

[30] Zou, F., X. Wang, X. Han, G. Rothschild, S. G. Zheng, U. Basu, J. Sun. 2018. Expression and function of tetraspanins and their interacting partners in B cells. Front. Immunol. 9: 1606. 30072987 10.3389/fimmu.2018.01606 PMC6058033

[31] Hauser, J. T., R. Lindner. 2014. Coalescence of B cell receptor and invariant chain MHC II in a raft-like membrane domain. J. Leukoc. Biol. 96: 843–855. 25024398 10.1189/jlb.2A0713-353R

[32] Friess, M. D., K. Pluhackova, R. A. Böckmann. 2018. Structural model of the mIgM B-cell receptor transmembrane domain from self-association molecular dynamics simulations. Front. Immunol. 9: 2947. 30619307 10.3389/fimmu.2018.02947 PMC6304377

[33] Gottwick, C., X. He, A. Hofmann, N. Vesper, M. Reth, J. Yang. 2019. A symmetric geometry of transmembrane domains inside the B cell antigen receptor complex. Proc. Natl Acad. Sci. USA 116: 13468–13473. 31209055 10.1073/pnas.1907481116 PMC6613136

[34] Grupp, S. A., K. Campbell, R. N. Mitchell, J. C. Cambier, A. K. Abbas. 1993. Signaling-defective mutants of the B lymphocyte antigen receptor fail to associate with Ig-alpha and Ig-beta/gamma. J. Biol. Chem. 268: 25776–25779. 8245014

[35] Mitchell, R. N., A. C. Shaw, Y. K. Weaver, P. Leder, A. K. Abbas. 1991. Cytoplasmic tail deletion converts membrane immunoglobulin to a phosphatidylinositol-linked form lacking signaling and efficient antigen internalization functions. J. Biol. Chem. 266: 8856–8860. 2026599

[36] Schamel, W. W., M. Reth. 2000. Stability of the B cell antigen receptor complex. Mol. Immunol. 37: 253–259. 10930632 10.1016/s0161-5890(00)00025-0

[37] Shaw, A. C., R. N. Mitchell, Y. K. Weaver, J. Campos-Torres, A. K. Abbas, P. Leder. 1990. Mutations of immunoglobulin transmembrane and cytoplasmic domains: effects on intracellular signaling and antigen presentation. Cell 63: 381–392. 2119890 10.1016/0092-8674(90)90171-a

[38] van Deventer, S. J., V. E. Dunlock, A. B. van Spriel. 2017. Molecular interactions shaping the tetraspanin web. Biochem. Soc. Trans. 45: 741–750. 28620035 10.1042/BST20160284

[39] Angelisová, P., I. Hilgert, V. Horejsí. 1994. Association of four antigens of the tetraspans family (CD37, CD53, TAPA-1, and R2/C33) with MHC class II glycoproteins. Immunogenetics 39: 249–256. 8119731 10.1007/BF00188787

[40] Spaargaren, M., E. A. Beuling, M. L. Rurup, H. P. Meijer, M. D. Klok, S. Middendorp, R. W. Hendriks, S. T. Pals. 2003. The B cell antigen receptor controls integrin activity through Btk and PLCgamma2. J. Exp. Med. 198: 1539–1550. 14610042 10.1084/jem.20011866 PMC2194118

[41] Hynes, R. O. 2002. Integrins: bidirectional, allosteric signaling machines. Cell 110: 673–687. 12297042 10.1016/s0092-8674(02)00971-6

[42] van Spriel, A. B., S. de Keijzer, A. van der Schaaf, K. H. Gartlan, M. Sofi, A. Light, P. C. Linssen, J. B. Boezeman, M. Zuidscherwoude, I. Reinieren-Beeren, 2012. The tetraspanin CD37 orchestrates the α_4_β_1_ integrin-Akt signaling axis and supports long-lived plasma cell survival. Sci. Signal. 5: ra82. 23150881 10.1126/scisignal.2003113

[43] Chiampanichayakul, S., A. Szekeres, P. Khunkaewla, S. Moonsom, V. Leksa, K. Drbal, G. J. Zlabinger, R. Hofer-Warbinek, H. Stockinger, W. Kasinrerk. 2002. Engagement of Na, K-ATPase beta3 subunit by a specific mAb suppresses T and B lymphocyte activation. Int. Immunol. 14: 1407–1414. 12456588 10.1093/intimm/dxf112

[44] Harton, J., L. Jin, A. Hahn, J. Drake. 2016. Immunological functions of the membrane proximal region of MHC class II molecules. F1000Res. 5 (F1000 Faculty Rev): 368. 10.12688/f1000research.7610.1 PMC4798158 27006762

[45] Stipp, C. S., T. V. Kolesnikova, M. E. Hemler. 2001. EWI-2 is a major CD9 and CD81 partner and member of a novel Ig protein subfamily. J. Biol. Chem. 276: 40545–40554. 11504738 10.1074/jbc.M107338200

