# Supplementary material for: Proximity-Based Labeling Identifies MHC Class II and CD37 as B Cell Receptor–Proximal Proteins with Immunological Functions
Source: Immunohorizons. 2024 Apr 16;8(4):326–38. doi: 10.4049/immunohorizons.2400014 (PMC11066716; doi:10.4049/immunohorizons.2400014)
Supplement: Supplemental Material_2 (PDF) [file IH_2400014_Supplemental_2.pdf]

Hoeger et al., Full Blots – Figure 2

Panel A

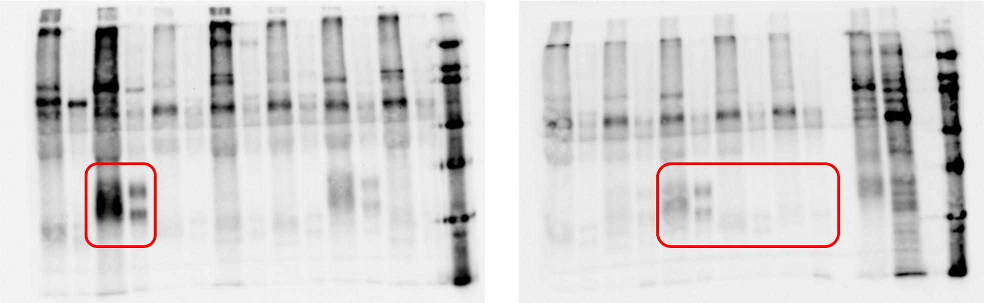

Panel B

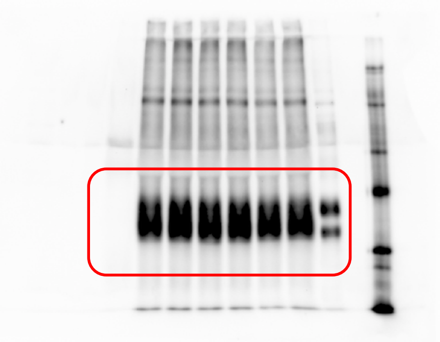

Panel C

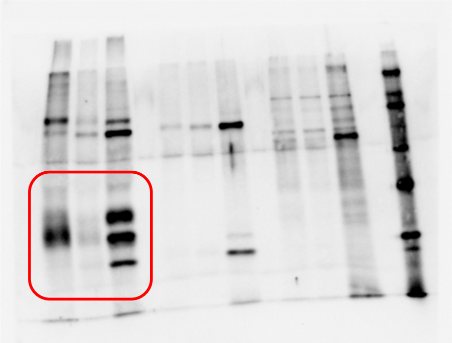

Panel D

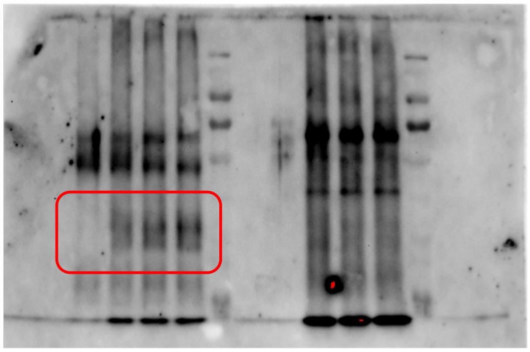

Red box indicates portion of blot included in final figure

Hoeger et al., Full Blots – Figure 3

Panel A

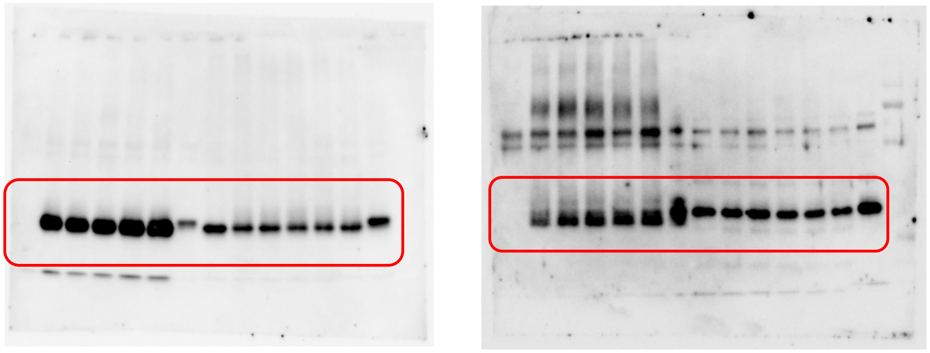

Panel B

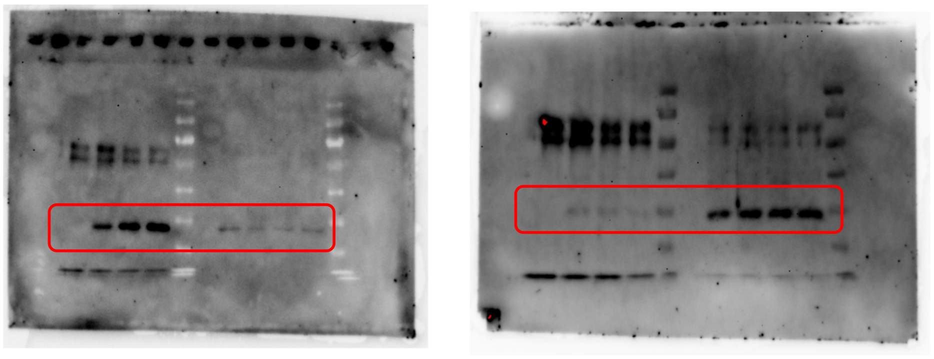

Red box indicates portion of blot included in final figure
